# Association between Reduction of Plasma Adiponectin Levels and Risk of Bacterial Infection after Gastric Cancer Surgery

**DOI:** 10.1371/journal.pone.0056129

**Published:** 2013-03-08

**Authors:** Hiroshi Yamamoto, Kazuhisa Maeda, Yoshitaka Uji, Hiroshi Tsuchihashi, Tsuyoshi Mori, Tomoharu Shimizu, Yoshihiro Endo, Aya Kadota, Katsuyuki Miura, Yusuke Koga, Toshinori Ito, Tohru Tani

**Affiliations:** 1 Department of Surgery, Shiga University of Medical Science, Shiga, Japan; 2 Department of Complementary & Alternative Medicine, Osaka University Graduate School of Medicine, Osaka, Japan; 3 Department of Health Science, Shiga University of Medical Science, Shiga, Japan; Northwestern University, United States of America

## Abstract

**Background and Purpose:**

Infections are important causes of postoperative morbidity after gastric surgery; currently, no factors have been identified that can predict postoperative infection. Adiponectin (ADN) mediates energy metabolism and functions as an immunomodulator. Perioperative ADN levels and perioperative immune functioning could be mutually related. Here we evaluated a potential biological marker to reliably predict the incidence of postoperative infections to prevent such comorbidities.

**Methods:**

We analyzed 150 consecutive patients who underwent elective gastric cancer surgery at the Shiga University of Medical Science Hospital (Shiga, Japan) from 1997 to 2009; of these, most surgeries (n = 100) were performed 2008 onwards. The patient characteristics and surgery-related factors between two groups (with and without infection) were compared by the paired *t*-test and χ^2^ test, including preoperative ADN levels, postoperative day 1 ADN levels, and ADN ratio (postoperative ADN levels/preoperative ADN levels) as baseline factors. Logistic regression analysis was performed to access the independent association between ADN ratio and postoperative infection. Finally, receiver operating curves (ROCs) were constructed to examine its clinical utility.

**Results:**

Sixty patients (40%) experienced postoperative infections. The baseline values of age, American Society of Anesthesiologists physical status, total operating time, blood loss, surgical procedure, C-reactive protein (CRP) levels, preoperative ADN levels, and ADN ratio were significantly different between groups. Logistic regression analysis using these factors indicated that type 2 diabetes mellitus (T2DM) and ADN ratio were significantly independent variables (*p<0.05, ** *p*<0.01, respectively). ROC analysis revealed that the useful cutoff values (sensitivity/specificity) for preoperative ADN levels, ADN ratio, blood loss, operating time, and CRP levels were 8.81(0.567/0.568), 0.76 (0.767/0.761), 405 g (0.717/0.693), 342 min (0.617/0.614), and 8.94 mg/dl (0.583/0.591), respectively.

**Conclusion:**

T2DM and ADN ratio were independent predictors of postoperative infection and ADN ratio was the most useful predictor for postoperative infection.

## Introduction

Infections are important postoperative complications following gastric surgery that result from complex interactions between age, gender, socioeconomic status, comorbidities, and local and systemic processes [1], [2]. Local factors include surgical technique and the degree of contamination, whereas systemic factors include pro- and anti-inflammatory cytokines that mediate postoperative inflammatory responses and can contribute to infection progression [3]. Serum levels of pro-inflammatory cytokines such as tumor necrosis factor-α (TNF-α), interleukin 6 (IL-6), and C-reactive protein (CRP) increase after surgery, especially with infectious complications [4], [5].

Adiponectin (ADN; also identified as apM1, ACRP30, AdipoQ, and GBP-28 in the literature) is an adipocyte-derived secretory protein that plays a key role in metabolism. Decreased ADN levels are associated with obesity and insulin resistance and are predictive factors of type 2 diabetes mellitus (T2DM), dyslipidaemia, and coronary artery disease [6]–[13].

In addition, ADN exhibits anti-inflammatory properties, as both animal and human studies have reported inverse associations between ADN and various inflammatory markers, including TNF-α, IL-6, and CRP levels [14]–[16]. Recently, we reported that plasma ADN levels decrease in rats with polymicrobial sepsis, indicating that an ADN deficiency triggered infection in the animal model [17]. Moreover, the relative decrease in preoperative ADN levels is reportedly a risk factor for postoperative infection [18]. These findings suggest that ADN might be a core factor in the early stages of human infections. In the present study, we examined the usefulness of the postoperative day (POD) 1/preoperative ratio of ADN levels as a prognostic factor of postoperative infection following abdominal surgery to design practical, simple, and specific interventions to prevent postoperative infections.

## Materials and Methods

### Patient population and study design

We analyzed 150 consecutive patients admitted to the Shiga University of Medical Science Hospital (Shiga, Japan), who underwent elective gastric cancer surgery from 1997 to 2009; of these, majority (100 cases) were performed from 2008 onwards. All patients received conventional and prophylactic antibiotic therapy for 3 days after surgery [19]. Blood samples were collected and ADN plasma levels were measured before surgery and on POD1 using a latex particle-enhanced turbidimetric assay (Otsuka Pharmaceutical Co., Ltd., Tokyo, Japan) [20]; in this assay, agglutination is generated using rabbit anti-human ADN polyclonal antibodies immobilized on latex beads through an antigen-antibody reaction. Briefly, 2 µL of plasma and 90 µL of reagent 1 (0.1 M Tris–HCl buffer containing 0.9% NaCl; pH 8.0) were injected into the reaction cuvette. After incubation for 5 min at 37°C, 90 µL of reagent 2 (antibody-immobilized latex bead suspension in 0.01 M Tris–HCl buffer; pH 8.0) was added to the cuvette to start the turbidimetric immunoreaction. After an additional 5 min, the ADN level was calculated from the difference in absorbance values between two time points (5 and 10 min) via dual-wavelength measurement (primary wavelength, 570 nm; secondary wavelength, 800 nm). The calibration curve was obtained from a series of working ADN calibrators and used to calculate the values of the plasma samples. The within- and between-run coefficients of variation (CV) were 08%–1.9% and 1.1%–2.0%, respectively, which were highly correlated with enzyme-linked immunosorbent assay–based methods (*r* = 0.99) [20]. In addition, the CRP levels were measured on POD1 as well.

Postoperative surgical site infections (SSIs) and remote infections were recorded during the hospitalization period. SSI was defined using the criteria of the Centers for Disease Control and Prevention (CDC; Atlanta, GA, USA) [21]. Superficial and deep incisional SSIs were characterized by the presence of purulent discharge from the incision site. Organ/space SSIs included anastomotic leakage and intra-abdominal abscesses and characterized by the presence of purulent discharge from a drain placed into the organ/space or an abscess involving an organ/space. Remote infections included those involving the respiratory, urinary, and gastrointestinal tracts and catheters.

This study conformed to the Clinical Research Guidelines of Shiga University of Medical Science and was approved by the institutional ethics committee. We obtained written informed consent to participate in this study from all patients.

### Statistical analysis

The patient characteristics and surgical data, including body mass index (BMI; mass in kg/height in m^2^), operation time (min), blood loss (g), and surgical methods (total or partial gastrectomy), were collected from patients' medical records [22]–[24]. The American Society of Anesthesiologists (ASA) physical scores were used to assess preoperative patient fitness: an ASA score of 1–2 was classified as low, whereas a score of 3–5 was classified as high [25]. Because patients with ASA scores of 4 and 5 are not suitable for surgery, only those with scores of 1–3 were included in this study.

Differences in patient characteristics and surgical factors between the two groups (those with infection and those without) were examined using the paired *t*-test for continuous variables and the χ^2^ test for dichotomized variables. A *p*-value of <0.05 was considered statistically significant.

Thereafter, the odds ratios (ORs) and 95% confidence intervals (CIs) for postoperative infection were estimated using four logistic regression models. Model 1 included basic potential risk factors [gender, age, BMI, log (blood loss), ASA, T2DM, and the surgical method]. In addition to the basic factors present in model 1, model 2 included CRP levels as well; model 3 included ADN ratio; model 4 included preoperative and levels; and model 5 included CRP and preoperative levels and ADN ratio. In these models, blood loss volume was included as a log transferred value [log (blood loss)] due to its skewed distribution, whereas CRP levels were not mathematically manipulated due to a near normal distribution.

The ORs for a continuous variable were expressed as that for one standard deviation (SD) increase in each variable. The ORs were only calculated for one SD decrease for ADN ratio.

Receiver operating curves (ROCs) were constructed for potential predictors of postoperative infection and the areas under the curves (AUCs) were calculated to determine the most useful cut-off values of ADN ratio, blood loss, time, and CRP levels, which were derived from the first derivative of the curve, and the sensitivity and specificity at the cut-off point were determined.

## Results

During the 14-day observation period, 60 patients (40%) experienced postoperative infections. Table 1 lists surgery-related risk factors and characteristics of patients with and without postoperative infections. Except the postoperative ADN levels, the baseline values of age, ASA, total operation time, blood loss, surgical procedure, CRP levels (POD1), preoperative ADN levels, and ADN ratio were significantly different between the two groups.

**Table 1:** Patient Characteristics.

	Infection(−) n = 90	infection(+) n = 60	p value
**Male Gender (%)**	67.8	73.3	0.467
**Age (years)**	64.8±11.8	68.6±11.0	0.047
**HT (cm)**	160.5±9.8	160.5±8.5	0.995
**BW (kg)**	57.0±10.9	55.8±11.9	0.515
**BMI**	22.0±3.2	21.6±4.0	0.491
**ASA (%)**			0.006
ASA1 (%)	39.5	15.5	
ASA2 (%)	55.8	74.1	
ASA3 (%)	4.7	10.3	
**History of diabetes mellitus (%)**	15.6	26.7	0.096
**Total operation time (min)**	325±98.2	399±122.5	<0.001
**Blood loss (g)**	366±363.0	858±782.4	<0.001
**Surgical procedure (total gastrectomy vs. partial gastrectomy) (%)**			<0.001
Total	22.2	53.3	
Partial	77.8	46.7	
**CRP (mg/dl)**	8.4±3.3	9.6±3.6	0.035
**Hb (g/dl)**	12.4±3.0	11.9±1.7	0.256
**Preoperative ADN (µg/ml)**	8.9±4.9	10.8±5.2	0.026
**Postoperative ADN (µg/ml)**	7.5±4.1	7.3±3.9	0.788
**ADN ratio**	0.86±0.2	0.67±0.1	<0.001

p: Difference in means or rates were tested using Student's t-test or Chi-square test.

HT, height; BW, body weight; BMI, body mass index; ASA, American Society of Anesthesiologists; CRP, C reactive protein; Hb, hemoglobin; AND, adiponectin.

Of the 13 patients who developed SSIs, three were incisional and 10 occurred in organs/spaces; however, 55 patients developed remote infections, including 36 cases of pneumonia, seven cases of enterocolitis, six catheter-related infections, three cases of sepsis, two cases of cholangitis, and one urinary tract infection. Eight patients developed more than one postoperative infection and one patient died of liver failure due to cholangitis. One of the 10 SSIs, in the patients who developed organ/space SSIs, was associated with anastomotic leakage. The presence of a postoperative infection was confirmed on mean postoperative day 5.8 (range, 1–21 days).

Logistic regression analyses were performed to identify independent risk factors for postoperative infection and the effect of preoperative plasma ADN levels was examined via this analyses. A history of T2DM was independently associated with postoperative infection in all models, although a significant difference was not identified in the univariate analysis (Table 1). CRP levels were revealed no significant association with infection in Model 2. As a result, while ADN ratio and preoperative ADN levels were independent variables in Model 3 and Model 4 in the logistic analyses, respectively, ADN ratio alone was a significantly independent variable when both ADN ratio and preoperative ADN levels were adjusted in Model 5. The OR for 0.2 decrease in ADN ratio was approximately 15 for Models 3 and 5.

**Figure 1:**
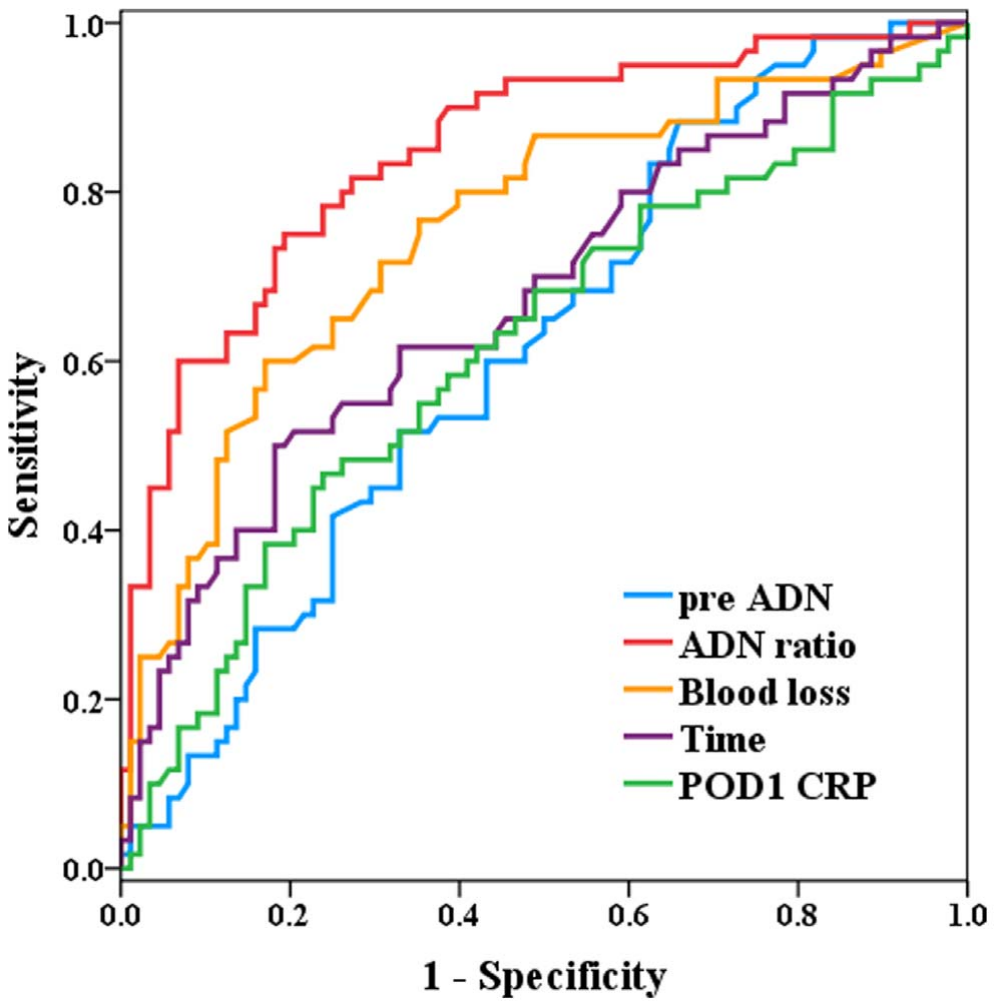
The receiver operating curves (ROCs) of postoperative infection for four potential predictors. The AUC values for preoperative ADN levels, ADN ratio, blood loss, operating time, and CRP levels were 0.616, 0.848, 0.757, 0.675, and 0.617, respectively. The useful cut-off values (sensitivity/specificity) for predicting preoperative ADN levels, ADN ratio, blood loss, operating time, and CRP levels were 8.81 µg/dL (0.567/0.568), 0.76 (0.767/0.761), 405 g (0.717/0.693), 342 min (0.617/0.614), and 8.94 mg/dL (0·583/0·591), respectively.

The ROCs indicated that ADN ratio was a better predictor of postoperative infection than preoperative ADN levels, blood loss, operation time, or CRP levels. The AUC for ADN ratio was 0.848. In addition, the useful cut-off values (sensitivity/specificity) for predicting preoperative ADN levels, ADN ratio, blood loss, operation time, and CRP levels were 8.81 µg/mL (0.567/0.568), 0.76 (0.767/0.761), 405 g (0.717/0.693), 342 min (0.617/0.614), and 8.94 mg/dL (0.583/0.591), respectively (Figure 1).

ROCs were similar by gender, age group, BMI, and total versus partial gastrectomy. Moreover, we did not find any significant correlation between gender, age, or BMI with ADN ratio and postoperative infection via logistic regression analyses (data not shown).

## Discussion

Our analyses of patient characteristics between those with and without postoperative infections showed that age, ASA, total operation time, blood loss, surgical procedure, CRP levels (POD1), preoperative ADN levels, and ADN ratio were significantly different between groups (Table 1). In the logistic regression analysis, ADN ratio was still significant even when both CRP levels and ADN ratio were included in Model 4, indicating that ADN ratio was an independent risk factor of postoperative infection following gastric cancer surgery. In addition, a history of T2DM was independently associated with postoperative infection in all models (Table 2), although a significant difference was not detected by univariate analysis (Table 1). CRP level was not an independent risk factor of postoperative infection. From the ROC curves, ADN ratio was a better predictor of postoperative infection compared with blood loss, operating time, or CRP levels (Figure 1).

**Table 2:** The multivariate adjusted odds ratios for postoperative infection.

Model	One SD	Model 1		Model 2		Model 3		Model 4		Model 5	
		Odds Ratio	95%CI	Odds Ratio	95%CI	Odds Ratio	95%CI	Odds Ratio	95%CI	Odds Ratio	95%CI
Male Gender		0.92	0.36–2.32	0.89	0.35–2.31	1.45	0.47–4.46	1.30	0.41–4.14	1.51	0.45–4.9
Age (years)	11.60	1.16	0.91–1.47	1.21	0.94–1.56	1.21	0.88–1.66	1.32	0.95–1.85	1.30	0.91–1.60
BMI (kg/m^2^)	3.80	0.81	0.54–1.23	0.80	0.52–1.21	0.89	0.557–1.39	0.88	0.56–1.38	0.91	0.58–1.41
ASA1 (%)			1(ref.)		1(ref.)		1(ref.)		1(ref.)		1(ref.)
ASA2 (%)		3.01	0.98–9.21	2.89	0.90–9.30	3.52	0.75–16.41	3.45	0.72–16.55	3.40	0.70–16.75
ASA3 (%)		3.66	0.59–22.80	3.60	0.55–23.62	2.75	0.30–25.47	3.00	0.31–29.15	2.60	0.30–26.40
DM (%)		3.50	1.11–11.05*	4.00	1.20–13.28	5.28	1.35–20.69	5.56	1.37–22.66	5.86	1.42–24.30
Total operation time	114.10	1.55	0.88–2.74	1.58	0.88–2.84	1.62	0.79–3.29	1.76	0.84–3.68	1.71	0.81–3.50
Log (blood loss)	616.20	6.59	1.88–23.14**	6.18	1.69–22.66**	2.83	0.70–11.52	2.54	0.60–10.80	2.86	3.60–12.82
Surgical procedure (%)		2.20	0.79–6.11	2.40	0.82–7.00	2.74	0.77–9.76	3.32	0.88–12.50	3.21	0.90–12.24
CRP (mg/dl)	3.50			1.51	0.93–2.43			1.52	0.86–2.67	1.53	0.90–2.71
ADN ratio	0.20					14.90	4.62–48.00	15.73	4.64–53.37**	15.20	4.50–51.13

* *p*<0.05,

** *p*<0.01.

Model 1: Basic potential risk factors [gender, patient age, BMI, log (blood loss), ASA, T2DM, and the surgical procedure]; Model 2: Basic factors plus CRP levels; Model 3: Basic factors plus ADN ratio; Model 4: Basic factors plus preoperative ADN levels; Model 5: Basic factors plus CRP and preoperative ADN levels and ADN ratio. Blood loss was included in the models as log (blood loss), which was normally distributed, whereas CRP levels were mostly normally distributed. The odds ratio for each continuous variable was expressed for one standard deviation increase [11.61 for age; 3.82 for BMI; 616.17 for log (blood loss); 114.12 for time; 3.47 for CRP levels (POD 1); 5.09 for preoperative ADN levels; and 0.21 for ADN ratio]. The odds ratio was calculated for one standard deviation decrease only for the ADN ratio. CI: confidence interval.

ADN is secreted by mature adipocytes; however, in contrast to leptin, lower ADN levels are associated with obesity, insulin resistance, diabetes, and disordered lipid metabolism [10]–[12], [26] and exhibits anti-inflammatory properties that inhibit monocyte adhesion and macrophage function [11]. The acute perioperative reduction in ADN levels, as observed in patients who subsequently developed infections, might be a contributing factor mediating disordered postoperative energy metabolism and a vigorous inflammatory response, which may contribute to a greater propensity for infection.

Recently, aggressive insulin-based perioperative glucose control was shown to improve patient outcome, including substantial reductions in infection rates [27]. It is likely that lowered ADN levels induce insulin resistance resulting in disordered glucose metabolism and subsequent infection. Hence, insulin therapy might counteract the dysfunction in energy regulation due to reduced ADN levels.

Although the mechanism for an acute reduction in ADN levels following surgery remains unknown, the plasma ADN levels in obese subjects were found to change only slightly despite significant diet-induced weight loss [28]. Several substances released into the circulation following surgery have been reported to suppress ADN expression, including inflammatory cytokines, such as IL-6 and TNF-α, hypoxia, reactive oxygen species (ROS), and counter-regulatory hormones including catecholamines and corticosteroids [26], [29], [30]. In the present study, the significant increase in postoperative IL-6 levels, a pro-inflammatory cytokine, in response to surgical stress, could account for the larger postoperative reduction in ADN ratios; this could be attributed to the fact that potent anti-inflammatory cytokines, including ADN, and pro-inflammatory cytokines have been proven to be antagonistic [11], [31]. Moreover, we performed logistic regression and ROC analyses for post-operative IL-6 and TNF-α levels; however, they were not important predictors of infection (data not shown).

An alternative explanation for decreased plasma ADN levels may be perioperative ADN protein binding, as lipoproteins have been shown to bind with lipopolysaccharides (LPS) during sepsis [32]. The decrease in high density lipoprotein cholesterol (HDL) observed during sepsis is related to the effect of LPS on a wide range of apolipoproteins, plasma enzymes, lipid transfer factors, and receptors involved in HDL metabolism. All lipoprotein classes have been shown to bind LPS and can attenuate the biological response of LPS *in vitro* and in rodents. We and other investigators recently observed that ADN binds to LPS to decrease ADN levels in rats with polymicrobial sepsis, with a reciprocal increase in TNF-α levels [17], [33]. LPS levels decrease in proportion to ADN levels. Although the underlying mechanisms are not entirely known, reduced ADN levels may result from LPS-ADN binding with subsequent sequestration. However, further studies are needed to better understand the mechanism behind the postoperative reduction in ADN levels.

Regarding the mechanism behind acute reduction in ADN levels following surgery, its significant association with blood loss disappeared when the ADN ratio was included in the logistic regression analysis in models 3 and 4 (Table 2). This might be attributable to a strong correlation between ADN ratio and blood loss (Table 2), as it is most likely that plasma ADN levels decreased with blood dilution, thereby suggesting that blood loss is a stronger independent risk factor for postoperative infection.

We concluded that a postoperative decrease in plasma ADN levels was not due to blood loss or blood dilution due to infusion because the ADN levels were unchanged after the removal of 400 ml of blood for autotransfusion (median blood loss among the 150 patients, 395 ml) (data not shown).

Factors associated with wound complications following elective gastrointestinal surgery included smoking, male gender, perioperative blood loss, and duration of surgical comorbidities [1], [2]. In the present study, we found that the incidence of postoperative infection was greater in patients who sustained a substantial reduction in postoperative ADN levels and this correlation remained after statistical corrections for the effect of blood loss and perioperative inflammation.

As a limitation to this study, we could not validate the significance of the predictors in a validation cohort because the total number of patients was too small.

If ADN is the underlying cause of disordered postoperative energy metabolism resulting in more serious infections, treatments with an ADN secretagogue are justified. The administration of ADN secretagogues increases ADN levels and improves insulin resistance [34]. Hyperglycemia and insulin resistance are common in critically ill patients, even if they did not have diabetes before. Intensive insulin therapy maintains blood glucose levels and reduces morbidity and mortality rates among critically ill patients in surgical intensive care units [22] and minimizes the effects of insulin resistance, thereby substantially improving postoperative outcomes [32].

The CDC guidelines for the prevention SSIs recommend the administration of surgical antibiotic prophylaxis for 24 h in clean-contaminated surgeries, such as gastrectomy [35]. In contrast, the duration of surgical antibiotic prophylaxis is 3–4 days in Japan [23]. A multicenter examination is currently being performed by the Japanese Society for Surgical Infection: this examination evaluates prophylactic antibacterial properties of surgical antibiotic administration over 24 h and for 4 days after several types of major surgeries, including total gastrectomy. Because the ADN ratio can be assessed within 1–2 days after surgery, it can be used to predict the necessity of continual administration of antibacterial agents to control and/or prevent infections.
